# Diversity, abundance of anopheline species, and malaria transmission dynamics in high-altitude areas of western Cameroon

**DOI:** 10.21203/rs.3.rs-5558659/v1

**Published:** 2025-01-14

**Authors:** Belinda Claire KIAM, Aline Gaelle TUEDOM BOUOPDA, Ibrahima IBRAHIMA, Samuel J WHITE, Pacôme K. TCHUENKAM, Zachary R. POPKIN-HALL, Mariama MBOUH, Jean Arthur MBIDA MBIDA, Charlène Tina NANSSONG, Luc Marcel ABATE, Clément Janvier ONGUENE, Brigitte FOTSO TUMAMO, Jacob M. SADLER, Jonathan B. PARR, Jessica T. LIN, Jonathan J. JULIANO, Innocent Ali MBULLI, Rhoel R. DINGLASAN, Sandrine Eveline NSANGO

**Affiliations:** University of Douala; University of Douala; University of Douala; University of North Carolina at Chapel Hill; University of Dschang; University of North Carolina at Chapel Hill; University of Dschang; University of Douala; University of Douala; MIVEGEC, Université Montpellier, IRD, CNRS; Centre Pasteur du Cameroun; Centre Pasteur du Cameroun; University of North Carolina at Chapel Hill; University of North Carolina at Chapel Hill; University of North Carolina at Chapel Hill; University of North Carolina at Chapel Hill; University of Dschang; University of Florida; University of Bertoua

**Keywords:** Anopheles, diversity, behavior, transmission, highland, western Cameroon

## Abstract

**Background:**

Assessing vector bionomics is crucial to improving vector control strategies. Several entomological studies have been conducted to describe malaria transmission in different eco-epidemiological settings in Cameroon; knowledge gaps persist, particularly in highland areas. This study aimed to characterize malaria vectors in three localities along an altitudinal gradient in the western region: Santchou (700 m), Dschang (1400 m), and Penka Michel (1500 m).

**Methods:**

Human landing catches were conducted from May to June 2023 from 6:00 pm to 9:00 am. Mosquitoes were sorted into genera, and all *Anopheles* species were identified using morphological taxonomic keys and species-specific Polymerase Chain reaction (PCR). Entomological indicators were assessed including species composition and abundance, biting behavior, infection rate, and entomological inoculation rate (EIR). Genomic DNA from the head and thoraces were tested for *Plasmodium*infection by real-time PCR.

**Results:**

2,835 Anopheles mosquitoes were identified, including *An. gambiae, An. coluzzii, An. funestus, An. leesoni, An. nili,* and *An. ziemanni*, with *An. gambiae* being the most prevalent at all sites. The human-biting rate of *An. gambiae s.l.* was significantly higher (p-value < 0.001) in Penka Michel compared to Santchou and Dschang (45.25 b/h/n vs 3.1 b/h/n and 0.41 b/h/n), and appears to be the most infected vector, and infectious vector distribution is highly focal, with entomological inoculation rates 13-fold higher in Penka Michel compared to Santchou (1.11 vs 0.08ibites/human/night). *P. falciparum* was the dominant malaria parasite (67% at Santchou, 62% at Penka Michel), but *P. malariae* (30%) and *P. ovale* (1.21%) infections were also detected.

**Conclusion:**

The study highlights a difference in mosquito composition and host-seeking behavior with altitude and the need for continued surveillance to monitor vector populations and prevent potential malaria outbreaks in these highland areas.

## Background

Malaria remains a major public health problem worldwide [1]. The latest WHO estimates point to 249 million cases and 608,000 deaths, with 94% of cases occurring in the African region. Children under 5 years and pregnant women represent the most vulnerable and affected groups. In addition to therapeutic care, considerable progress has been made in the implementation of vector control measures such as long-lasting insecticidal nets (LLINs) and indoor residual spraying (IRS) [2,3]. The rapid spread of these strategies has so far been a major factor in the decline in malaria morbidity and mortality over the past two decades [3].

Malaria remains a major public health challenge in Cameroon, in 2023, Cameroon recorded 4.85 million cases of suspected malaria, of which over a million were severe, mainly in children under 5. Malaria contributed to 7.3% of hospital deaths [4]. The entire population of approximately 28 million people is at risk of malaria, with the East, Centre, and North regions being the most affected [5]. The intensification of free LLIN distribution has contributed substantially to reducing the malaria burden in the country, with an estimated population coverage of 80% [5–7]. However, the effectiveness of these control tools is threatened by several factors, including insecticide resistance, changes in the feeding and resting behavior of the main malaria vectors [8–12], and the spatiotemporal variation in malaria transmission across different ecological zones of the country. Cameroon is often known as “Africa in miniature” due to the diversity of its natural environment distributed across its 10 provinces: deserts, coasts, mountains, rainforests, and savannah, resulting in heterogenous conditions [13] that directly influence mosquito distribution, abundance, survival, and malaria parasite development.

Variations in vector ecology across the landscape can lead to uneven exposure and transmission risks [14]. The lowlands are characterized by floods and swamps, promoting larvae survival and a greater abundance of vectors, resulting in high malaria prevalence [15–17]. In contrast, the highlands consist of hills and valleys, resulting in a sparse distribution of larval habitats and a lower malaria prevalence (*P. falciparum* prevalence based on RDT positivity of 11–19%) [18]. The diverse malaria eco-epidemiological contexts and local vector ecologies call for tailored interventions for each transmission focus, as malaria epidemiology fluctuates over time and is correlated with the local success of control programs.

The success of these control strategies therefore depends on up-to-date information on bionomic, and vector transmission patterns in different epidemiological contexts, particularly in regions at high risk of epidemics, including high-altitude areas. In recent years, mountainous regions of Africa have experienced an increase in the frequency and intensity of malaria epidemics. These areas, characterized by lower temperatures and humidity, present unique challenges for malaria transmission [19–22]. Unlike holoendemic lowland regions, highland areas are typically hypoendemic, making successful entomological inoculation more likely [23–25]. Despite the significance of the Bamileke plateau in western Cameroon (large population and role in the country’s economic life), research on malaria transmission in these areas has been limited [26,27]. Moreover, recent reports of *Plasmodium vivax* infections in Dschang [28,29] further underscore the need for a comprehensive understanding of malaria dynamics in this region. To address this knowledge gap, we conducted a series of entomological and parasitological (unpublished data) surveys in three localities of the region. We aimed to assess the extent of malaria transmission and identify the primary vectors driving the disease, considering the region’s diverse ecological settings and population movements.

## Materials and methods

### Description of the study area

**Figure 1:**
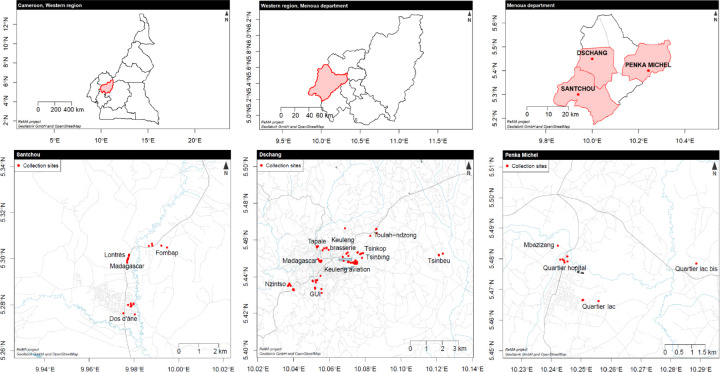
Map showing the study sites in the Western Region of Cameroon.

The study was carried out from May to June 2023 in three high-altitude localities in the Menoua division along an altitudinal transect in the Western region of Cameroon: Santchou (700m), Dschang (1400m) and Penka Michel (1500m) are located in a savannah landscape within the Guineo-Congolese bioclimatic domain, on the volcanic line of Cameroon [30,31] (Fig. 1).

Santchou (5°25’N; 9°58’E) is located at an altitude of 700m in the vast Mbô plain, bounded to the south by the Manengouba mountain and to the north by a sloping cliff, delimiting it from the Foréké village. The area is mesoendemic for malaria, with a prevalence of 36.19% [27]. Santchou covers an area of 335 km^2^ and has a population ranging from 15,000 to around 25,000 and benefits from a dense hydrographic (the river Nkam and its tributaries) network and good rainfall. As a semi-urban city, Santchou has a low plain topography with a hygromorphic soil type. The climate is equatorial Guinean, characterized by four seasons of unequal duration: the long rainy season (mid-August to October); the short rainy season (March to June); the long dry season (mid-October to March); and the short dry season (June to mid-August). Average rainfall is 1,363.3 mm/year and annual temperature ranges from 20.83°C to 23.35°C [32].

The town of Dschang (5°25’N; 10°03’E) is an urban environment that lies at the summit of the Mbô cliffs on the southeastern slopes of the Bamboutos mountains. It is bounded on the south by the commune of Santchou and on the northeast by the Bamboutos mountains. It covers an area of 262 km^2^ and has a population of 120,207 inhabitants. The topography consists of alternating hills and valleys crisscrossed by small watercourses. It is characterized by 2 seasons: a rainy season (eight months) from mid-March to mid-November and a dry season (four months) from mid-November to mid-March. The area is characterized by a mosaic of agricultural landscapes and is classified as hypoendemic for malaria, with a prevalence of 25.04% [27]. Average precipitation is 2,000 mm/year and the daily temperature range can exceed 23°C during the dry season which constitutes a particularity of this locality [33]

Penka Michel (5°28’N; 10°15’E) landscape is marked by a shallow topography, with a population of 98,229 inhabitants and a population density of 383.7 inhabitants/km². The area has a relatively low malaria prevalence of 38.4% among children under five years old [34]. The relief is that of an undulating plateau with a flat bottom. As in Dschang (29 km from Penka Michel), the area is characterized by a sub-equatorial climate, with distinct wet and dry seasons. The rains peak in August-September when up to 345.1 mm of rainfall can be recorded monthly. Temperatures are relatively low and constant due to the high altitude, with an annual temperature range of only 26°C [35].

These diverse ecological settings, ranging from lowland to highland, provide an ideal opportunity to study the influence of environmental factors on malaria transmission dynamics. This study was conducted following the fourth distribution campaign of Long-Lasting Insecticidal Net (LLIN) in the Western region of Cameroon in 2022.

### Mosquito collections and processing

Households were randomly selected in each site to assess malaria transmission using the human-landing catch (HLC) approach [36] from 6:00 pm to 9:00 am.

In Dschang, HLCs were carried out in 10 villages in four health zones (Bafou, Fialah-foreke, Foto, and Siteu). Four and three villages were selected respectively for mosquito collection in Santchou and Penka Michel.

During each survey, adult mosquitoes were collected indoors (living room or bedroom) and outdoors (outdoor shelter) in randomly selected homes, with the distance between two houses being at least 100m. HLCs were carried out during 11 nights in Dschang with a total number of 166 men/nights, 5 nights in the Santchou district with a total number of 78 men/nights, and 4 nights in the Penka Michel district with a total number of 50 men/nights leveraging trained volunteers. To minimize collectors’ bias due to the variability of attractiveness and collection skills, volunteers changed positions every two hours between indoor and outdoor stations and rotated through the houses. Each collector was provided with a flashlight and a bag containing hemolysis tubes with cotton. Mosquitoes landing on the legs of the collectors were captured using haemolysis tubes and stored in a labeled bag. At each sampling station, bags containing mosquitoes were collected hourly for morphological identification.

### Anopheles larval collection and identification of adult mosquitoes

Anopheles larval surveys were carried out in all the water collections identified in the study areas. The pre-imaginal stages of anopheles present in these sites were collected using the Dipping method [37] and brought back to the insectarium for rearing. On arrival at the insectarium, the larvae were transferred to tanks containing water from the lodge, according to their stage of development, and then fed with TetraMinBaby®. The resulting pupae were transferred to small cups and placed in adult-rearing cages until they emerged.

Mosquitoes collected were sorted by genus (*Culex, Anopheles, Aedes, Mansonia,* and others) and species level using specific identification keys [38]. *Anopheles* mosquitoes were then dissected into head-thorax, and abdomen, preserved individually in Eppendorf tubes containing silica gel for further molecular analysis.

### Laboratory processing of mosquitoes

#### DNA extraction, molecular identification of *Anopheles* species

Genomic DNA was extracted from the head-thorax and legs using a 2% CTAB (cetyltrimethylammonium bromide) solution according to Collins *et al* [39]. Genomic DNA was amplified by multiplex Polymerase Chain Reaction (PCR) and targeted the intergenic spacer of the ribosomal DNA (rDNA IGS) and internal transcribed spacer region 2 (ITS2) of *An. gambiae s.l.* and *An. funestus s.l*, respectively [40,41]. Subsequently, restriction fragment length polymorphism (RFLP) was performed for *An. gambiae s.l* speciation [40].

#### Detection of *Plasmodium-infected* mosquitoes

Genomic DNA extracted from *Anopheles* head-thorax was tested for the presence of *Plasmodium spp.* by real-time PCR according to Mangold *et al* [42]. The primer sequences target a polymorphic fragment of the small subunit of 18S ribosomal RNA (18S rRNA), enabling the speciation of *P. falciparum, P. vivax, P. ovale,* and *P. malariae.*

### Statistical analysis

Data were recorded in a Microsoft Excel spreadsheet (Office 2016) and imported to R studio software (San Diego, CA, USA) for analysis. Entomological indices (including vector diversity, relative abundance, aggressiveness, biting cycle and behavior, endophagy, and infection rate (IR) were calculated and presented as descriptive statistics. The chi-square test was used to compare mosquito density between sites. T-test and two-way ANOVA followed by Bonferroni’s multiple comparison tests were used to compare indoor and outdoor biting rates within the sites. The level of statistical significance was set at p-value ≤ 0.05.

Biodiversity was assessed using two common diversity indices: the Shannon-Weaver index (H’) [43] and the Simpson index (λ) [44]. These indices consider both the number of species (species richness) and the relative abundance of each species (evenness) in a sample.


$$H\prime =-\sum_{i=1}^{S}p_{i}\log\left(p_{i}\right),\;\lambda =\sum_{i=1}^{S}p_{i}^{2}\;\text{and}\;\lambda =1-D$$

The similarity between the species communities at the three sites (Santchou, Dschang, and Penka Michel) was assessed using two ecological metrics: the General Jaccard Index [45], calculated as $\text{GJI\%}\;=\;\text{d}/\left(\text{a}\;+\;\text{b}\;+\;\text{c}\;-\;2\text{d}\right)$, where: a = number of species unique to community Santchou; b = number of species unique to community Dschang; c = number of species common to all the three communities; d = number of species unique of Penka Michel and the Renyi diversity profile (H_α_) [46].


$$H_{a}=\frac{\text{ln}\left(\sum_{i=1}^{S}p_{i}^{\alpha }\right)}{1-\alpha }\left(\alpha =0;\;0.25;\;0.5;\;1;\;2;\;4;\;8;\;\infty \right)$$

The entomological inoculation rate (EIR) was calculated using the following formula:

EIR=IR×HBR, where IR= rate of female anopheles testing positive for *Plasmodium spp* infection, HBR= the human-biting rate expressed as the ratio of the number of anopheles mosquitoes captured by HLC to the total number of men by night.

## Results

### Diversity and specific richness of Culicidae fauna along the transect

A total of 7,935 mosquitoes belonging to six genera were collected over 294 person-nights. The distribution of collected mosquito genera by sampling site is shown in Table 1. Mosquitoes of the genus *Culex spp* were predominant (40.9%; n=3,245/7,935), while the genus *Anopheles* accounted for 39.82% (n= 3,160/7,935). In addition to these genera*, Aedes, Mansonia, Coquillettidia,* and *Eretmapodie* were also collected, representing respectively 5.11% (n= 406/7,935), 14.13% (n= 1,122/7,935), 0.05% (n= 4/7,935) and 0.01% (n= 1/7,935). Overall, more mosquito was collected in Penka Michel (41.11%, n=3,262/7,935) compared to Dschang (33.23%, n=2,637/7,935) and Santchou (25.62%, n=2,033/7,935) and the difference in the distribution of mosquitoes between sites was statistically significant (p-value=0.0001, X^2^=9671, ddl=10). The genus *Anopheles* was more prevalent in Penka Michel (86.91%, n= 2,835) while in Dschang *Culex spp* represented 94.25% (n= 2,493) of the collection, and Santchou, *Mansonia spp* was predominant at 55.2%, (n= 1,119).

**Figure 2:**
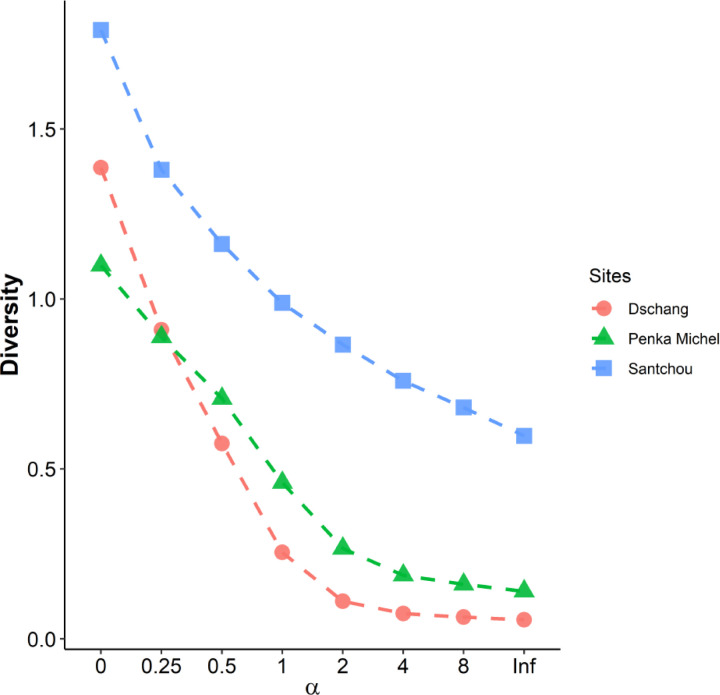
Diversity profiles based on Rényi’s H-alpha series between the three sites

Analysis of the Shannon-Weaver diversity indices (H’) reveals a clear pattern: Santchou boasts the highest diversity (H’ = 1.028), followed by Penka Michel (H’ = 0.41) and Dschang (H’ = 0.263). By using the Simpson diversity index (D), where lower values indicate higher diversity. Santchou (D = 0.219) has the lowest D value, followed by Penka Michel (D = 0.904) and lastly Dschang (D = 0.992). These findings suggest a relatively even distribution of species abundance in Santchou, while Dschang is dominated by *Culex* mosquitoes and Penka Michel by *Anopheles*. Further analysis using Jaccard’s similarity index (60%) indicates a moderate level of similarity among the three communities. This observation is corroborated by the Renyi alpha biodiversity profiles depicted in Fig 2. These profiles suggest that while each community likely harbors a dominant species, other species are present in suficient numbers to contribute to overall diversity.

### *Anopheles* species composition and abundance

A total of 3,448 Anopheles mosquitoes were collected during the entomological survey, comprising 3,160 adults and 288 immature stages (larvae and pupae). Immature stages were primarily found in stagnant water pools. The limited availability of suitable anopheles breeding sites likely contributed to the lower number of larvae collected (75 in Dschang, 98 in Santchou, and 115 in Penka Michel). All immature stages were identified morphologically. *An. gambiae s.l.* was the only species found in breeding sites.

**Figure 3:**
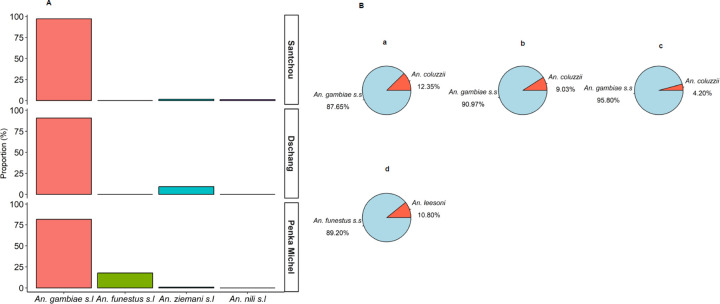
Anopheles fauna and abundance according to the study site; A: Global species composition B: Species composition of *Anopheles* sibling species; a=species composition within the *An. gambiae* complex in Santchou; b=species composition within the *An. gambiae* complex in Dschang; c=species composition within the *An. gambiae* complex in Penka Michel; d=species composition within the *An. funestus* group in Penka Michel.

Among the 3,160 adult mosquitoes, four species were morphologically identified: *An. gambiae sl. (82.88%)*, *An. funestus s.l. (15.92%), An. nili (0.09%)* and *An. ziemanni* (1.11%). Fig.3.A shows the proportion of *Anopheles* species collected at each site throughout the study. *An. Gambiae s.l.* was the predominant vector in all sites representing 82.9% (2,619/3,160), while *An. ziemanni,* which is considered a secondary malaria vector represented only 1.61%, 9.21%, and 0.85% in Santchou, Dschang, and Penka Michel, respectively.

Molecular speciation revealed that *An. gambiae* and *An. coluzzii* were the only members of the *An. gambiae* complex identified in the three study sites. *An. gambiae* was the main malaria vector, with 87.6% (n=298/340), 90.1% (n=131/144), and 95.8% (n=365/381), respectively, in Santchou, Dschang, and Penka Michel. For a subset of 176 *An. funestus s.l*. analyzed, 89.2% (n=157/176) were *An. funestus s.s.* and 10.8% (n=19/176) were *An. leesoni* (Fig. 3. B)

No significant difference was recorded in the diversity of different anopheline mosquitoes across the study area. However, based on Shannon Weaver diversity indices for *Anopheles* species (Santchou H’=0.99, Dschang H’=0.25, and Penka Michel H’=0.33), Santchou stands out as the site with the greatest species diversity, harboring a rich array of species with a relatively balanced distribution of abundances.

### *Anopheles* biting behavior and cycle.

**Figure 4:**
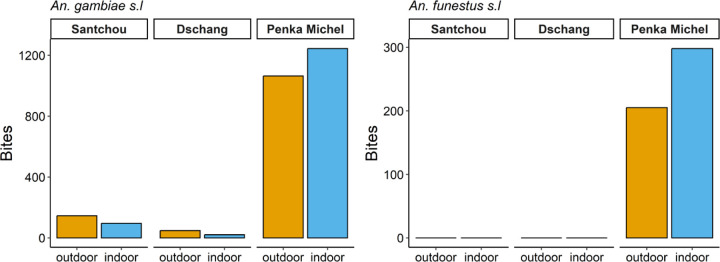
*An. gambiae s.l.* and *An. funestus s.l.* biting behavior in Santchou, Dschang, and Penka Michel

*Anopheles* mosquitoes were collected outdoors and indoors and the relative aggressiveness (bites/human/night) of the dominant vectors *An. gambiae s.s.* and *An. funestus s.s.* is shown in Fig.4. *Anopheles* mosquitoes bit both outdoors and indoors, with a significant difference observed in Dschang (64.47% *vs* 35.53%; p-value=0.039), Santchou (59.84% *vs* 40.16%; p-value=0.196), and Penka Michel, (54.50% *vs* 45.50%; p-value=0.577), with the tendency towards exophagy in Dschang.

**Figure 5:**
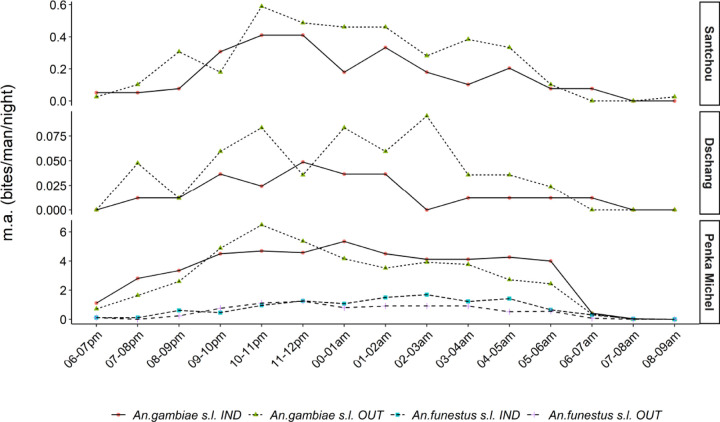
Biting cycles of An. gambiae s.l. and An. funestus s.l. collected indoors and outdoors in Santchou, Dschang, and Penka Michel.

According to the biting cycle (Fig. 5), the major malaria vectors were captured both indoors and outdoors during all collection periods (from 6:00 p.m. to 9:00 a.m.). However, there were notable differences across sites. In Santchou, *An. gambiae s.l.* biting activity varied throughout the night, starting to increase gradually at 08:00 p.m., peaking between 10:00 to 11:00 p.m. both indoors and outdoors, and a gradual decrease until 09:00 a.m. In Dschang, mosquito bites occurred mostly outdoors with a peak between 2:00 to 3:00 a.m. outdoors and between 11 p.m. to 12:00 p.m. indoors. In Penka Michel, *An. gambiae s.l.* biting activity was intense in the first half of the night, peaking between 10:00–11:00 p.m. outdoors and between 12:00 p.m.−1:00 a.m. indoors. For *An. funestus s.l*, the biting activity increased progressively, with high biting activity in the early hours of the morning, between 2:00 a.m.−3:00 a.m. indoors.

### *Plasmodium* infection rate

Of the 720 *Anopheles* mosquitoes tested by real-time PCR for the presence of *Plasmodium* infections. Only 19 (2.7%) of the mosquitoes were found to be infected by *Plasmodium*. These mosquitoes were from three species (*An. gambiae, An. funestus,* and *An. ziemanni*). In Santchou, the overall infection rate was 2.4% (6/249), with *An. gambiae* carrying mono-infections of *P. falciparum* at 66.67% (4/6) and *P. malariae* at 33.33% (2/6), respectively. In Dschang, no infected specimen was detected among the 76 mosquitoes analyzed. In Penka Michel, the overall infection rate was 3.2% (13/392). *An. gambiae, An. funestus,* and *An. ziemanni* tested positive for *P. falciparum at* 61.54% (8/13;)*, P. malariae* at 30.76% (4/13;), and *P. ovale* at 7.7% (1/13;).

### Human biting rate and entomological inoculation rate

The entomological inoculation rate (EIR) in each study site is presented in Table 2. *An. gambiae s.l.* was the most aggressive species with a human biting rate (HBR) of 3.1, 0.41, and 45.25 bites per human per night (b/h/n) respectively in Santchou, Dschang, and Penka Michel (p-value=0.0001). The global EIR for Penka Michel was 1.84 infective bites per human per night (ib/h/n), 23 times higher than in Santchou (0.08ib/h/n). The level of malaria transmission varies between *Anopheles* species and sites; *An. gambiae* appears to be the most competent malaria vector with an estimated EIR of 0.08ib/p/n in Santchou, and 1.1ib/p/n in Penka Michel, followed by *An. funestus* with 0.47 ib/p/n. The contribution of *An. ziemanni* in the transmission was recorded in Penka Michel, with 0.04 ib/p/n.

## Discussion

Entomological monitoring of malaria vectors plays a critical role in understanding their biology, ecology, and spatial distribution [47]. This knowledge is essential for designing targeted control strategies and deploying the most effective tools. This study was focused specifically on the highlands of Cameroon, where malaria control remains a challenge. Following the mass distribution of Long-lasting insecticidal nets (LLINs) as a vector control method, this study provides valuable data on key entomological parameters of malaria transmission in this region.

Six *Anopheles* vectors were identified: *An. gambiae s.s*, *An. coluzzii*, *An. funestus s.s*, *An. leesoni*, *An. nili*, and *An. ziemanni*. This observed diversity is consistent with the varied aquatic habitats present in the study area, including permanent and temporary water bodies such as streams, lakes, rivers, and puddles, which provide suitable breeding grounds for these malaria vectors. However, a notable decline in overall species diversity compared to a previous study by Tchuinkam et *al*. [31] suggests potential shifts in the local mosquito population; these findings align with previous studies in the same region [48,49]. Climate change and associated environmental alterations are likely contributing factors to these changes, as reported for other Cameroonian epidemiological contexts [7]. The presence of these vectors, particularly the highly anthropophilic and competent *An. gambiae s.s.* and *An. funestus s.s* suggest their critical role in the persistence of malaria transmission in the study area.

Among the *Anopheles* species identified, *An. gambiae* was the most prevalent across all three study sites. This dominance aligns with previous findings in Cameroon’s West Region [31,50,51]. Its adaptability to highland environments is also well documented [5,52]. *An. funestus*, another major vector, was only found in Penka Michel at 1500m, representing 17.7% of the anopheline population collected. Only three specimens of *An. nili s.l*. were collected in Santchou and *An. ziemanni* was present at very low densities. This uneven distribution of *Anopheles* species likely stems from several factors, with temperature differences playing a potential role. During the rainy season, the temperatures are lower at Penka Michel and Dschang compared to the lowland site (Santchou) might explain some variation in vector densities. Previous studies have shown that cooler temperatures can delay larval development and increase mortality [53–55]. However, Penka Michel, despite having a similar climate to Dschang and located at roughly the same altitude, exhibited densities of *An. gambiae* thirty-three times higher. These observations suggest that climatic influence may not fully explain the discrepancies in vector densities. The hilly topography of Penka Michel likely plays a significant role. This geography promotes water accumulation and retention, creating more abundant and stable breeding sites for *Anopheles* species (swamps, rivers, lakes). Balls *et al.* (2004) demonstrated a link between flat-bottomed highland valleys, prone to water accumulation, and increased malaria risk in Tanzania. Similarly, other studies [56,55,52] highlight altitude, topography, and land use as significant environmental factors impacting malaria vector abundance in highland regions [57].

The observed differences in vector densities between Penka Michel and Dschang, despite similar altitudes, can be also attributed to factors such as urbanization. Dschang, with its higher level of urbanization, exhibited a significantly higher abundance of *Culex* spp (alone accounted for 76.8% of the total *Culex* spp. collected). This finding aligns with previous observations in the city [49,48], which reported a high abundance of larval habitats and populations of *Culex* spp. The *Culex* genus is an indicator of urbanization [48], whose predominance in urban areas could be attributed to factors such as intense human activity (economic and industrial), and inadequate environmental management practices, which can create suitable breeding sites [58,59]. While *Culex* spp. dominated the urban landscape, and in rural areas, the distribution changes. *Aedes* spp. and *Mansonia* spp. were also present, reaching 10.3% and 55.2% in Penka Michel and Santchou, respectively. These genera represent a substantial public health concern due to their established roles as vectors for various diseases in Africa [60]. *Aedes* spp., for instance, are notorious for transmitting arboviruses such as dengue and yellow fever, while *Culex* spp., *Anopheles* spp., and *Mansonia* spp. are implicated in the transmission of lymphatic filariasis [36–38]. Specific to our study, we identified four *Aedes* species: *Ae. simpsoni*, *Ae. albopictus*, *Ae. aegypti*, and *Ae. africanus*. These species have been associated with the transmission of dengue and other arboviral diseases in various regions [64–66], underscoring the potential public health risks posed by their presence in the study area.

Consistent with previous studies in the west region [31,52,51], this study identified the presence of both *An. gambiae s.s.* and *An. coluzzii*, sibling species within the *An. gambiae s.l*. complex, co-existing along the altitudinal transect (sympatry). *An. gambiae s.s*. is known to be more adapted to semi-urban environments, preferring sunlit larval habitats. Conversely, *An. coluzzii* typically thrives in urbanized areas with artificial, polluted, and wetter breeding sites [51,67,68]. Furthermore, the *An. funestus* group taxa found exclusively at Penka Michel were primarily composed of *An. funestus s.s*. and *An. leesoni*. Recently the epidemiological role of *An. leesoni* in malaria transmission was demonstrated in certain regions of Cameroon [69].

An interesting observation was the difference in *An. gambiae s.l*. biting behavior across the altitudinal transect. Compared to previous studies [31] reporting endophagic behavior (biting indoors) in the same area, this study found exophagic behavior (feeding outdoors) in Dschang and exophilic behavior (resting outdoors) in Santchou. This shift could be attributed to behavioral changes in response to intensified vector control efforts, particularly the widespread distribution of LLINs: on this date, the household coverage rate in Milda is 100 (unpublished data). Similar trends have been observed in recent Cameroonian studies [10,52] and across Africa [70–72]. However, continuous entomological monitoring is crucial to understand better these mosquito species’ spatial and behavioral variations in the study sites. In Penka Michel, both *An. gambiae* and *An. funestus* exhibited primarily endophagic behavior. This might be linked to the high mosquito nuisance levels, prompting residents to spend more nights indoors, which influences mosquito-biting behavior. Nevertheless, further investigations are needed to assess the sensitivity of these mosquito populations to the effects of insecticide-treated nets.

Taken as a whole, *An. gambiae* was aggressive throughout the night, both indoors and outdoors, with peak biting activity occurring between 10:00 pm and 3:00 am. This extended biting period could be linked to the relatively low long-lasting insecticidal net (LLIN) usage in the western region (58.7%), falling below the 80% coverage recommended by the WHO [73]. These findings align with previous studies, which reported peak biting activity for both species occurring late at night (between 00:00 pm and 6:00 am) [74,50,51]. It is important to acknowledge the limitations of the study. While we have assessed the impact of LLINs on malaria transmission, we were unable to fully evaluate the distribution and actual use of LLINs in our study sites. Further research is needed to address these gaps.

Overall, malaria transmission intensity across the study sites appeared low, with an entomological inoculation rate (EIR) ranging from 0.08 to 1.84 infected bites per human per night. Interestingly, *An. gambiae*, which was the most prevalent mosquito species biting exhibited the highest infection rate, followed by *An. funestus* and *An. ziemanni*. These findings contrast with the high EIR reported by Tchuinkam in 2010 [31] in the same area. This difference could potentially be attributed to climatic changes observed in recent years, where such variations have been shown in laboratory studies to affect survival and reproductive capacity [10].

Among the *Plasmodium* species identified, *P. falciparum* was the dominant parasite, accounting for up to 63% of infections in both Santchou and Penka Michel. This aligns with findings from other studies [25,75–77] and confirms the primary epidemiological role of *P. falciparum* in the region. Infections with *P. malariae* and *P. ovale* were also detected in infected vectors (*An. gambiae*, *An. funestus,* and *An. ziemanni*). The presence of non-falciparum malaria parasites in these locations highlights the importance of including these parasites in control programs to achieve successful malaria elimination efforts.

While no *P. vivax* infections were detected in the analyzed mosquito samples, this doesn’t necessarily exclude its presence in this region. Seminal studies have reported *P. vivax* cases in Duffy-negative individuals residing in the western Cameroon region (Dschang, Santchou), with prevalence ranging from 0.5% to 35% [28,29]. Furthermore, another study identified *P. vivax* infections within the *An. coluzzii* vector population in Tibati (Adamaoua region) [78]. This finding suggests the potential circulation of the parasite among the human population and its possible transmission by *Anopheles* mosquitoes. Additionally, Ossè and collaborators reported cases of *P. vivax* infection in vectors *An. gambiae* and *An. coluzzii* from Benin [79].

## Conclusion

We report on the variation in the abundance and dynamics of malaria vector populations in the West Cameroon highlands. Six *Anopheles* species were present in the study area, all known to be malaria vectors in Cameroon. *An. gambiae* was the most widespread species in the sites, followed by *An. funestus* although the distribution of the latter was highly focal. The uneven distribution of anopheline species within the study area further confirms that the presence of these species varies according to the micro and macro-environmental differences present in the bio-ecological zones, even at the same altitude. *An. gambiae has* shown a preference for biting outdoors rather than indoors in the lowland plain and on the mountain plateau, which could compromise malaria control strategies using mainly LLINs. Therefore, the implementation of additional tools (i.e., larviciding, integrated management, and environmental management) to combat these outdoor-biting mosquitoes must be considered.

## Figures and Tables

**Table 1:** frequency and diversity of Culicidae fauna in Dschang, Santchou, and Penka Michel

Species	Santchou		Dschang		Penka Michel	
	N	%	N	%	N	%
*An. gambiae s.l*	242	11.90	71	2.70	2,308	70.75
*An. funestus s.l*	-	-	-	-	503	15.42
*An. ziemanni*	4	0.20	7	0.27	24	0.74
*An. nili s.l*	3	0.15	-	-	-	-
*Ae.africanus*	0	0	51	1.93	320	9.81
*Ae. simpsoni*	0	0	3	0.11	0	0
*Ae. aegypti*	2	0.09	2	0.07	1	0.03
*Ae. palpalis*	4	0.19	5	0.18	0	0
*Ae. albopictus*	0	0	3	0.11	1	0.03
*Aedes spp.*	2	0.09	1	0.03	5	0.15
*Culex spp.*	652	32.07	2,493	94.54	100	3.07
*Mansonia spp.*	1,119	55.04	3	0.11	0	0
*Coquilletidia spp.*	4	0.2	0	0	0	0
*Eretmapodie spp.*	1	0.05	0	0	0	0
**Total**	**2,033**	**100**	**2,637**	**100**	**3,262**	**100**
Species richness	6		4		3	
Shannon_H	1.028		0.263		0.41	
Simpson_D	0.219		0.992		0.904	
Jaccard	60%					

N= number; %= percentage; p-value=0.0001, chi-square=9671, ddl=10

**Table 2:** The entomological inoculation rate (EIR) for each site.

Sites	Mosquito species	Tested	Positive	IR (%)	HBR (b/h/n)	EIR (ib/h/n)
**Santchou**	*An. gambiae s.s.*	242	6	2.5	3.1	0.08
	*An. funestus s.l.*	0	0	0	0	0
	*An. ziemanni*	4	0	0	0.05	0
	*An. nili s.l.*	3	0	0	0.03	0
	Global	249	6	2.41	3.19	0.077
**Dschang**	*An. gambiae s.s.*	69	0	0	0.41	0
	*An. funestus s.l.*	0	0	0	0	0
	*An. ziemanni*	7	0	0	0.04	0
	*An. nili s.l.*	0	0	0	0	0
	Global	76	0	0	0.45	0
**Penka Michel**	*An. gambiae s.s.*	285	7	2.45	45.25	1.11
	*An. funestus s.l.*	83	4	4.82	9.86	0.47
	*An. ziemanni*	24	2	8.33	0.47	0.04
	*An. nili s.l.*	0	0	0	0	0
	Global	392	13	3.32	55.58	1.84

IR (Infection Rate); EIR (Entomological Inoculation Rate); HBR (Human Biting Rate); p-value 0.05

## Data Availability

Data are archived and available on request from the corresponding author

## Abbreviations

CTAB: Cetyltrimethyl ammonium bromide
EIR: Entomological inoculation rate
IR: Infection rate
IRS: Insecticide residual spraying
LLINs: Long-lasting insecticidal nets
HBR: Human-biting rate
PCR-RFLP: PCR-restriction fragment length polymorphism
rRNA: Ribosomal ribonucleic acid
PNLP: National malaria control program
